# Femoral Large Bore Sheath Management: How to Prevent Vascular Complications From Vessel Puncture to Sheath Removal

**DOI:** 10.1161/CIRCINTERVENTIONS.124.014156

**Published:** 2024-08-21

**Authors:** Lazzaro Paraggio, Francesco Bianchini, Cristina Aurigemma, Enrico Romagnoli, Emiliano Bianchini, Andrea Zito, Mattia Lunardi, Carlo Trani, Francesco Burzotta

**Affiliations:** Department of Cardiovascular Sciences, Fondazione Policlinico Universitario A. Gemelli IRCCS, Rome, Italy (L.P., C.A., E.R., M.L., C.T., F. Burzotta).; Department of Cardiovascular and Pulmonary Sciences, Università Cattolica del Sacro Cuore, Rome, Italy (F. Bianchini, E.B., A.Z., C.T., F. Burzotta).

**Keywords:** femoral artery, heart assist device, peripheral artery disease, transcatheter aortic valve replacement, vascular access devices

## Abstract

Transfemoral access is nowadays required for an increasing number of percutaneous procedures, such as structural heart interventions, mechanical circulatory support, and interventional electrophysiology/pacing. Despite technological advancements and improved techniques, these devices necessitate large-bore (≥12 French) arterial/venous sheaths, posing a significant risk of bleeding and vascular complications, whose occurrence has been related to an increase in morbidity and mortality. Therefore, optimizing large-bore vascular access management is crucial in endovascular interventions. Technical options, including optimized preprocedural planning and proper selection and utilization of vascular closure devices, have been developed to increase safety. This review explores the comprehensive management of large-bore accesses, from optimal vascular puncture to sheath removal. It also discusses strategies for managing closure device failure, with the goal of minimizing vascular complications.

Despite technological advancements and optimized techniques, transfemoral large-bore arterial access (LBAA) and large-bore venous access (LBVA; ≥12 French [Fr]) are nowadays required for numerous percutaneous procedures, such as structural heart interventions, percutaneous mechanical circulatory support, and interventional electrophysiology/pacing.^1,2^ These devices pose a significant risk of bleeding and vascular complications, whose occurrence has been related to worse clinical outcomes.^3,4^ According to the latest VARC-3 (Valve Academy Research Consortium) definitions,^5^ access-related vascular complication is defined as any adverse clinical event potentially linked to the access site and include vascular closure device (VCD) failure. Therefore, effective management of vascular access is key to reducing VCs and improving clinical outcomes.^6^ This review explores the comprehensive management of large bore accesses, from optimal vascular puncture to sheath removal. It also discusses strategies for managing VCD failure, with the goal of minimizing VCs.

## FEMORAL ARTERY PUNCTURE

**Figure 1.**
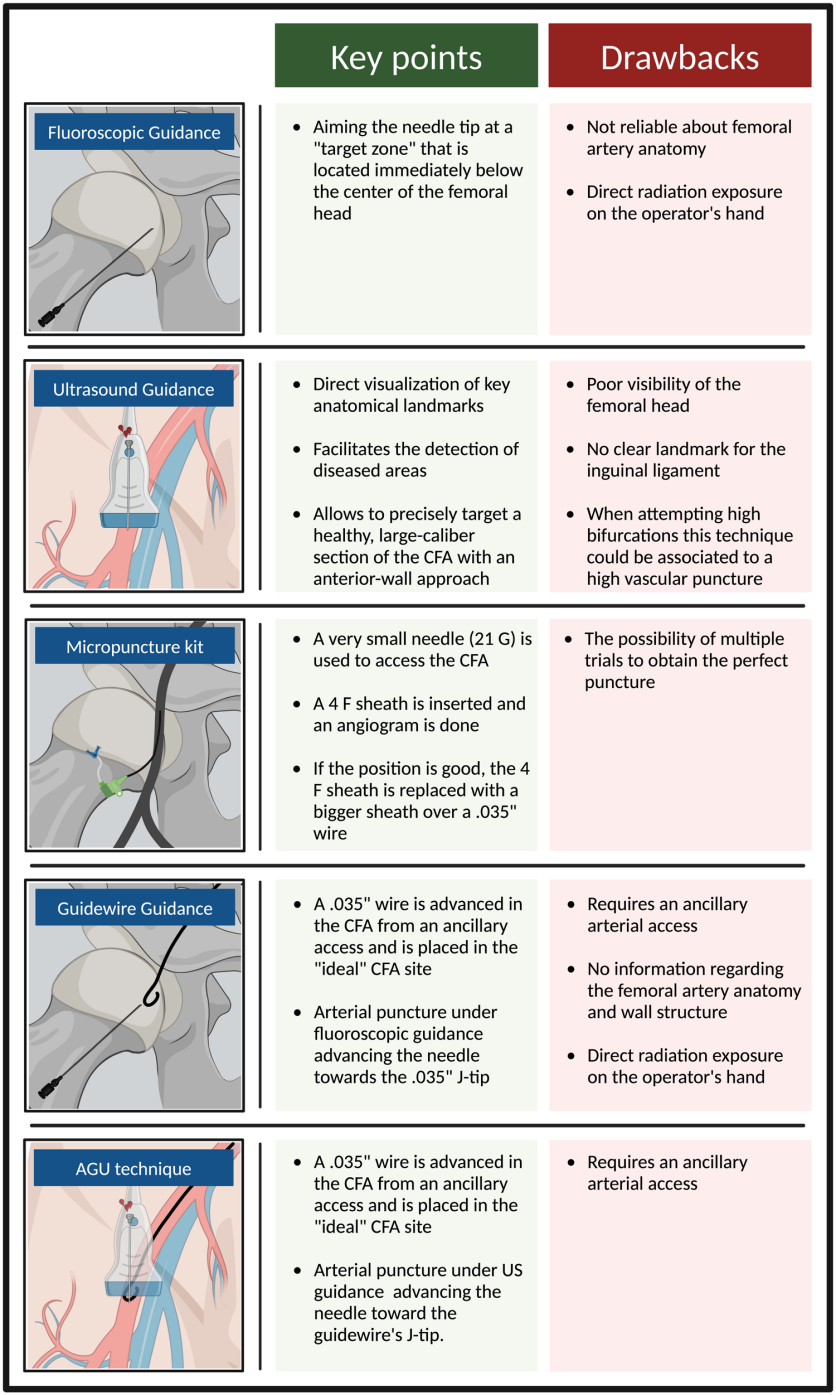
**Overview of common techniques for femoral artery puncture.** CFA indicates common femoral artery; and US, ultrasound. Created with BioRender.com.

**Figure 2.**
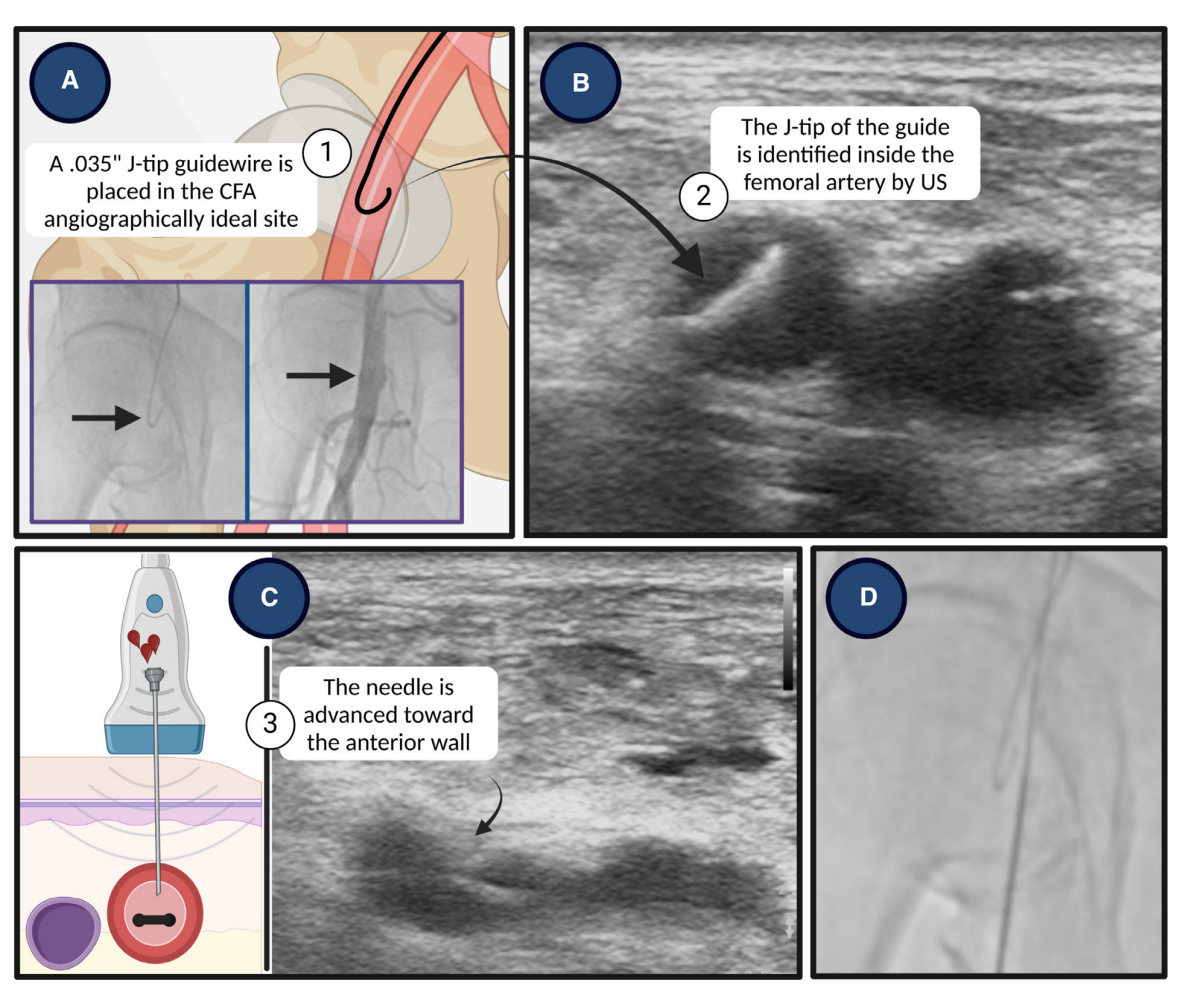
**The angio-guided-ultrasound (AGU) guidance.** CFA indicates common femoral artery; and US, ultrasound. Created with BioRender.com.

Typically, the common femoral artery (CFA) is identified as the optimal puncture site, owing to its considerable diameter and the ease of compressing it against the femoral head.^1^ The ideal target zone is below the inferior epigastric artery (which generally originates above the inguinal ligament) and above the artery bifurcation.^7^ It is well established that vascular complication rate increases in suboptimal CFA punctures.^8^ Puncturing below the bifurcation (ie, entering the superficial or profunda femoral arteries) increase the risk of hematoma, pseudoaneurysm, and arteriovenous fistula.^7^ Conversely, puncturing above the femoral head and inferior epigastric artery increases the risk of retroperitoneal hemorrhage.^7^ Several techniques can assist CFA puncture (Figure 1).^9^ Recently, a Micropuncture kit (Cook Medical, Bloomington, IN) has been introduced; it is designed to allow access to the CFA with a smaller needle (21-Gauge [G]; outer diameter [OD], 0.82 mm) as compared with the classic one (18-G; OD, 1.27 mm) to minimize bleeding and vessel trauma.^1,10^ Some studies showed that the use of Micropuncture kit, compared with a standard approach, reduced the rate of access-site bleeding and VCs.^11,12^ Concerning the best approach for CFA puncture, US guidance, compared with a traditional approach, resulted in fewer attempts and shorter time to access,^13^ a decreased likelihood of vein-puncture^13,14^ and reduced VCs.^15^ In addition, when a VCD is used, US guidance is associated with fewer major bleeding or VCs, as revealed by a subgroup analysis of the randomized clinical trial (RCT) UNIVERSAL (The Routine Ultrasound Guidance for Vascular Access for Cardiac Procedures)^16^ (odds ratio [OR], 0.61 [95% CI, 0.39–0.94]) and by a meta-analysis (d’Entremont et al; OR, 0.44 [95% CI, 0.23–0.82]).^17^ Besides advantages, US guidance could be associated with a high puncture when approaching high bifurcations. To mitigate this risk, checking needle position with fluoroscopy after US evaluation can be beneficial. Moreover, the previously described angio-guided-ultrasound technique^1,18^ may be used; it integrates multiple methods (Figure 2) to improve the accuracy of CFA puncture and requires ancillary arterial access (eg, radial).

## HEMOSTASIS TECHNIQUES BASED ON ELECTIVE VCD USE

Effective femoral-LBAA hemostasis is essential for reducing VCs and increasing overall procedural success.^19^ The routine use of VCDs has been shown to decrease the time required for hemostasis enhancing patient recovery, although it does not significantly reduce bleeding.^20^ Currently, in the United States, Food and Drug Administration (FDA) has approved the suture-based Perclose ProGlide/ProStyle (Abbott Vascular, Santa Clara, CA) and the plug-based MANTA device (Teleflex, Morrisville, NC) for femoral-LBAAs.^20^ These VCDs are classified as active approximation devices because they close the artery entry-point mechanically, through a suture (ie, ProGlide) or a mechanical plug (ie, MANTA).^20^ Two RCTs compared these VCDs in managing femoral-LBAAs. In the CHOICE-CLOSURE trial (Randomized Comparison of Catheter Based Strategies for Interventional Access Site Closure during Transfemoral Transcatheter Aortic Valve Implantation),^21^ when compared with ProGlide, MANTA was associated with a higher rate of in-hospital VARC-2 major/minor-VCs (relative risk [RR], 1.61 [95% CI, 1.07–2.44]; *P*=0.029) and access-site/access-related bleedings (RR, 1.58 [95% CI, 0.91–2.73]; *P*=0.133), but time to hemostasis was significantly shorter (80 [32–180] versus 240 [174–316] seconds; *P*<0.001). In the MASH trial (MANTA Versus Suture Based Vascular Closure After Transcathter Aortic Valve Replacement),^22^ there was no significant difference in the primary end point of VARC-2 access site–related VCs (10% versus 4%; *P*=0.16) and clinically significant access-site bleedings (9% versus 6%; *P*=0.57) between MANTA and ProGlide.

### Suture-Based VCD and Preclosure Techniques

**Figure 3.**
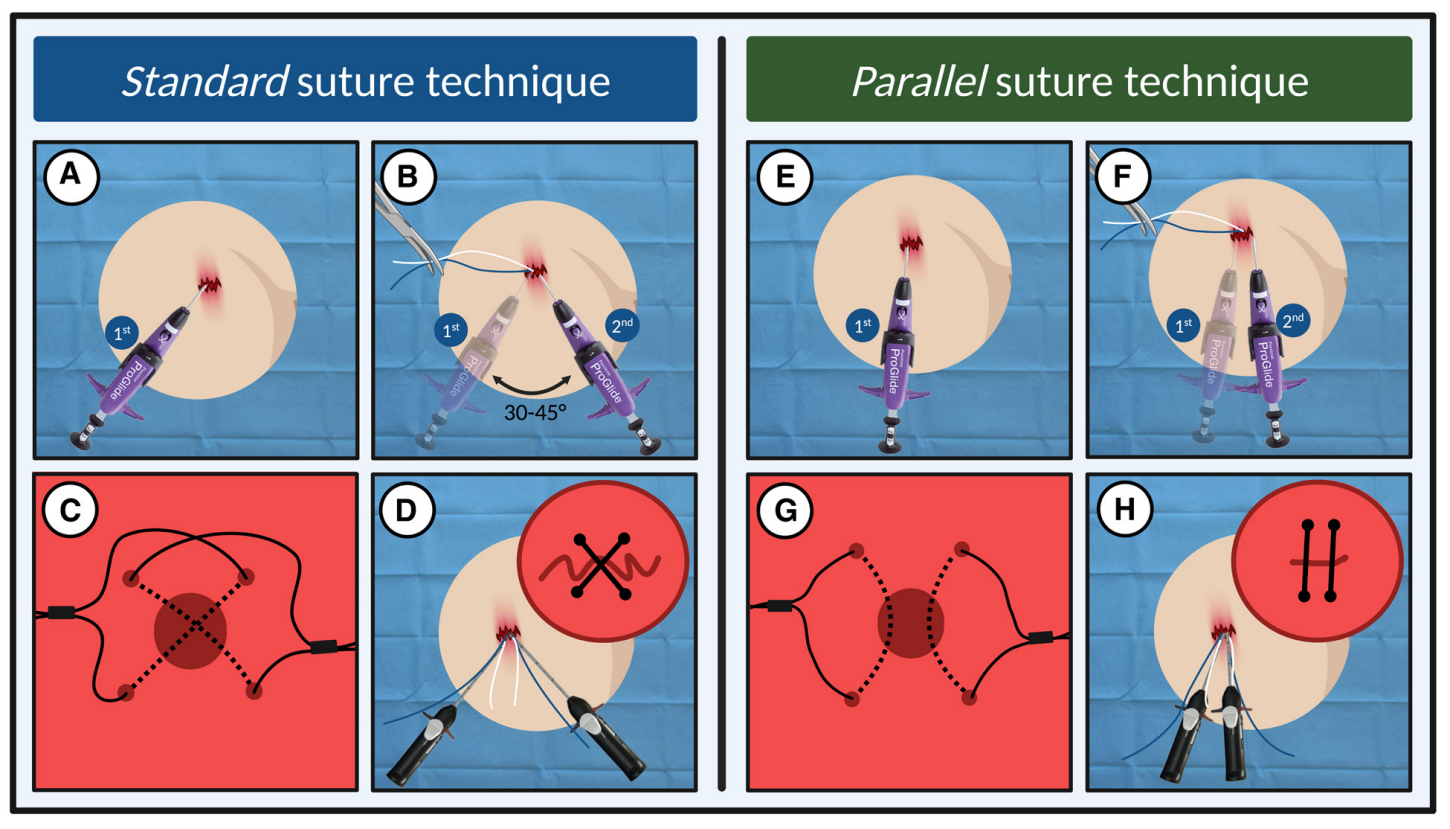
**Standard suture technique vs parallel suture technique.** Two Perclose ProGlide sutures are inserted into the vessel wall using the standard suture technique (**A** and **B**) or the parallel technique (**E** and **F**). After tightening the knots, crosswise (**C** and **D**) or parallel (**G** and **H**) orientation of the sutures from the intravascular view is shown. Created with BioRender.com.

ProGlide is suitable for arterial sheaths ranging up to 26-Fr OD. The device is positioned over a 0.035′′ wire, and intravascular placement is confirmed by pulsatile blood return from the marker lumen. Upon deployment, its footplate anchors inside the lumen against the vessel wall, and a needle forms a suture loop. Hemostasis is achieved by tightening the sutures using a dedicated sliding knot.^20^ According to the preclosure technique, the 3-0 polypropylene suture is deployed around the arteriotomy at the beginning of the procedure, and knot advancement is placed on hold until the procedure is complete.^23^ During the procedure, the sutures are secured with mosquito forceps or sterile plasters. For arteriotomies >8-Fr, it may be advisable to preimplant 2 ProGlide (double preclosure technique^24^; Figure 3). This implicates sequential insertion of 2 ProGlide devices at 30° to 45°, creating an interrupted X-figure closure.^25^ Although effective, this technique carries a risk of suture interference, potentially leading to device failure or femoral artery shrinkage.^25^ To address these concerns, a parallel preclosure technique has been described,^26^ where 2 ProGlide sutures are deployed parallel to the vessel on either side of the puncture site, after being moved medially and laterally (Figure 3). Despite its effectiveness, ProGlide use can be challenging in cases of high atherosclerotic burden or complex femoral anatomy; moreover, calcified plaques may hinder needle advancement and suture anchoring to the vessel wall. According to the large meta-analysis (>1500 patients) by Al-Abcha et al,^27^ the rate of ProGlide failure ranges around 10%. Additionally, to facilitate the management of eventual leaks, it is advisable to maintain a 0.035′′ guidewire and perform an angiography through the ancillary access (when available) before knot tightening; if required, a third suture-based (not advisable in small vessels/mild stenoses) or a plug-based VCD can be implanted.^28^ When an ancillary access is absent (eg, single-access Impella protected-PCIs), final angiographic check could be performed through a 4-Fr catheter (typically a Judkins right) that is introduced into the CFA and advanced into the distal descending aorta.^29^ Once positioned, ProGlide are secured around the catheter. Using digital subtraction imaging, the arterial axis is checked for any bleeding; if required, through a 0.035′′ guidewire an additional VCD is deployed.

### Plug-Based VCD

**Figure 4.**
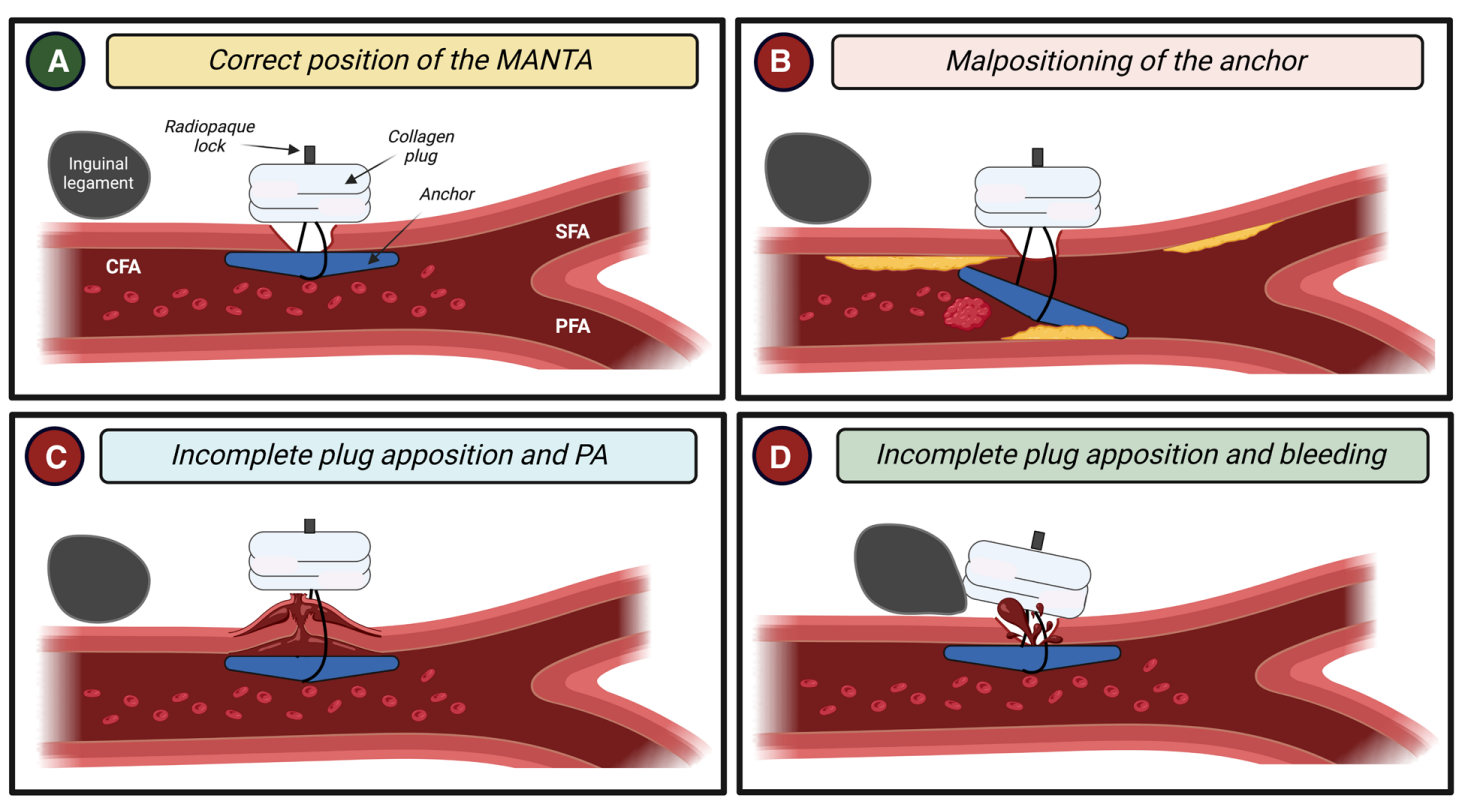
**MANTA-related vascular complications.** Correct placement of the MANTA device (**A**). Toggle dislodgement, often caused by severe artery calcification, can result in stenosis or acute occlusion (**B**). Inadequate placement of the toggle, which may occur due to a high puncture site and interference with inguinal ligament, can lead to minor/major bleedings (**C**). Improper apposition of the plug, often due to insufficient movement down the tampered tube, can cause pseudoaneurysm and potential late rupture (**D**). CFA indicates common femoral artery; PFA, profunda femoral artery; and SFA, superficial femoral artery. Created with BioRender.com.

MANTA is designed for femoral-LBAAs (12−25 Fr OD) without requiring preimplantation maneuvers. This device includes an 8-Fr depth locator, a 14/18-Fr sheath, an introducer, and a delivery handle.^30^ The delivery handle contains a closure unit with an extraluminal bovine collagen plug and an intraluminal bioabsorbable polymeric anchor made of polylactic-co-glycolic acid (the toggle). These components are connected by a suture and locked together using a small stainless steel clip creating a sandwich on both sides of the arteriotomy site. After the procedure, the sheath is replaced for the dedicated MANTA sheath over a stiff guidewire. The closure unit is then inserted and adjusted to the preset deployment depth (puncture depth plus 2 cm as assessed with the locator). Subsequently, the toggle is released, the assembly component retracted, and the collagen plug fixed to the exterior arterial wall with a stainless steel lock. Finally, distal perfusion must be verified either by angiography through an ancillary access or Doppler ultrasound. Once hemostasis is achieved, the guidewire is removed, and the suture is trimmed at the skin level. According to the large meta-analysis by Al-Abcha et al,^27^ the rate of MANTA failure can be estimated to be 7%. A recent study^31^ evidenced a higher incidence of VCs when the arteriotomy depth was ≥40 mm and CFA diameter <8 mm. MANTA failure is mainly attributed to some key mechanisms^32^ (Figure 4). (1) Toggle dislodgement, often caused by severe artery calcification, can result in stenosis or acute occlusion. (2) Inadequate toggle placement, which may occur due to a high puncture site and interference with the inguinal ligament, can lead to significant bleeding. (3) Improper plug apposition, often due to insufficient movement down the tampered tube, can cause pseudoaneurysm and potential late rupture. In cases of MANTA failure/complications and residual bleeding it is not possible to proceed with further VCD positioning; considering these aspects, having an ancillary access may facilitate the rapid deployment of balloons/stents.

### Mixed Techniques

Initially conceived as a solution for excessive bleeding, the combination of a suture-based and an additional collagen plug-based VCD might minimize CFA strain/constriction, while retaining the advantages of both types of VCDs. Various combinations have been described:

- The double preclose approach plus a plug-based VCD (Angio-Seal; Terumo Corp, Tokyo, Japan) in case of failure, demonstrated no significant differences in VARC-2 major/minor-VCs and bleedings when compared with the standard double preclose strategy.^33^
- A strategy combining a single preimplanted ProGlide at the beginning and the plug-based FemoSeal system (Terumo Corp, Tokyo, Japan) at the procedure’s end showed a reduced incidence of VARC-3 major-VCs or greater than or equal to type 2 bleedings, compared with a diagonally deployed double preclose strategy (at 10 and 2 o’clock).^34^ However, the study’s primary limitation, as a single-center retrospective analysis, is that the observed difference in outcomes was predominantly due to minor bleedings and device failures, which necessitated unplanned vascular surgery for newly developed femoral artery stenosis.
- Recently, the MULTICLOSE algorithm^35^ for LBAA closure after transcatheter aortic valve implantation has been described. In this approach, a preclosure with 1-2 suture-based VCDs is performed. At the procedure end, angiographic control via the primary access site with a 6 to 8 Fr sheath is performed to define a tailored final hemostatic strategy, which may be attempted with an additional suture- and plug-based VCD or none. Reported major VCs rate applying MULTICLOSE algorithm is <1%.^35^

## HEMOSTASIS TECHNIQUES IN THE ABSENCE OF ELECTIVE PRECLOSURE

**Figure 5.**
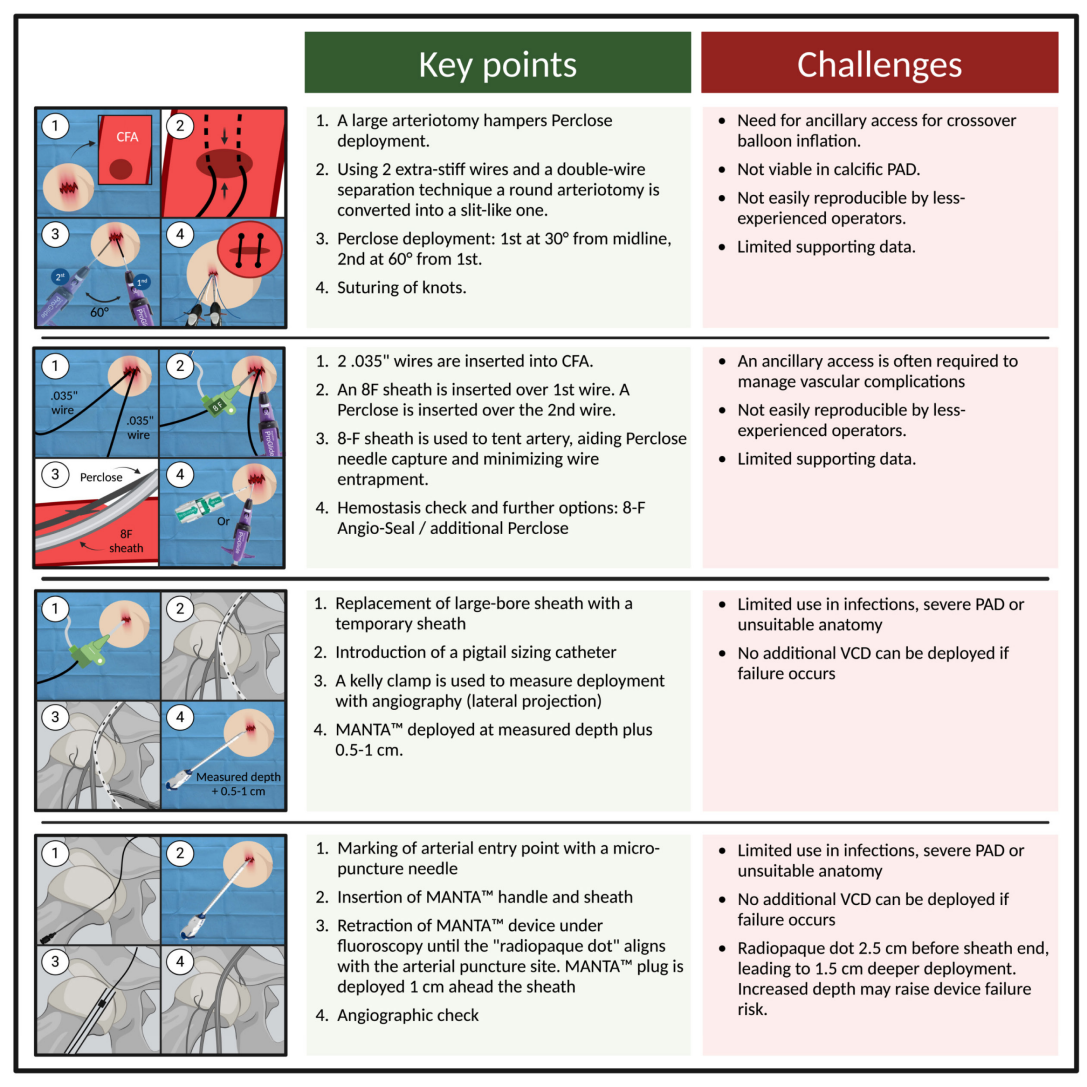
**Overview of hemostasis techniques in the absence of elective preclosure.** CFA indicates common femoral artery; PAD, peripheral artery disease; and VCD, vascular closure device. Created with BioRender.com.

When the large-bore sheath is implanted on emergency (ie, percutaneous mechanical circulatory support for cardiogenic shock/cardiac arrest) and in case of planned prolonged insertion, the preclosure methods are not feasible and it is crucial to have alternative solutions, avoiding manual compression alone; therefore, a VCD is advised. However, postprocedural closure of large-bore arteriotomies using ProGlide presents challenges due to its limited capacity for securing adequate anterior wall tissue.^36^ Thus, alternative approaches^37,38^ (described in Figure 5) have been described. In addition, the dry-seal technique may be applied^39^ by advancing a balloon from the contralateral femoral access (or through an ancillary transradial access) in the ipsilateral external iliac artery. The large-bore sheath may be then exchanged for a 45 to 55 cm long one; next, sheath is removed after balloon inflation and ProGlide deployment. The inflation is regulated to facilitate hemostasis, and, if residual bleeding occurs, the balloon is advanced to the arteriotomy site (ie, crossover balloon occlusion technique).

Although MANTA does not require preclosure, it typically necessitates the use of a specific locator to measure the arteriotomy depth before sheath insertion. To address this issue, recently, the manufacturer has introduced a specific 14-French depth locator that could be used to localize the arteriotomy depth even after the procedure. Therefore, when facing with larger bore sheaths (18-Fr) or in emergency setting, 2 alternative methods have been proposed to deploy MANTA: the Pigtail^40^ and the DOT technique^41^ (Figure 5).

## BAIL-OUT STRATEGIES IN CASE OF VASCULAR COMPLICATIONS

**Figure 6.**
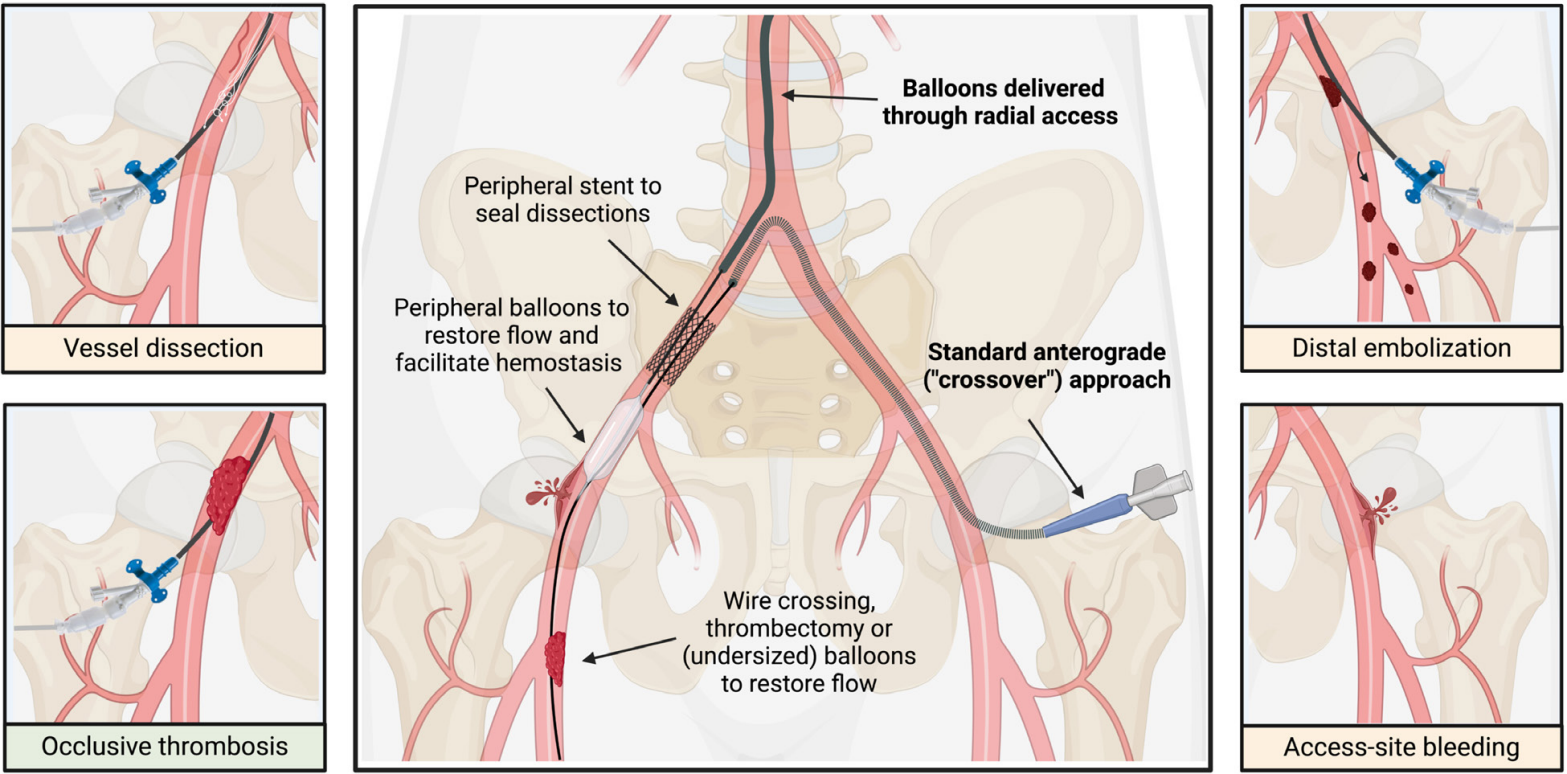
**Overview of most common vascular complications and endovascular bail-out management.** Created with BioRender.com.

**Figure 7.**
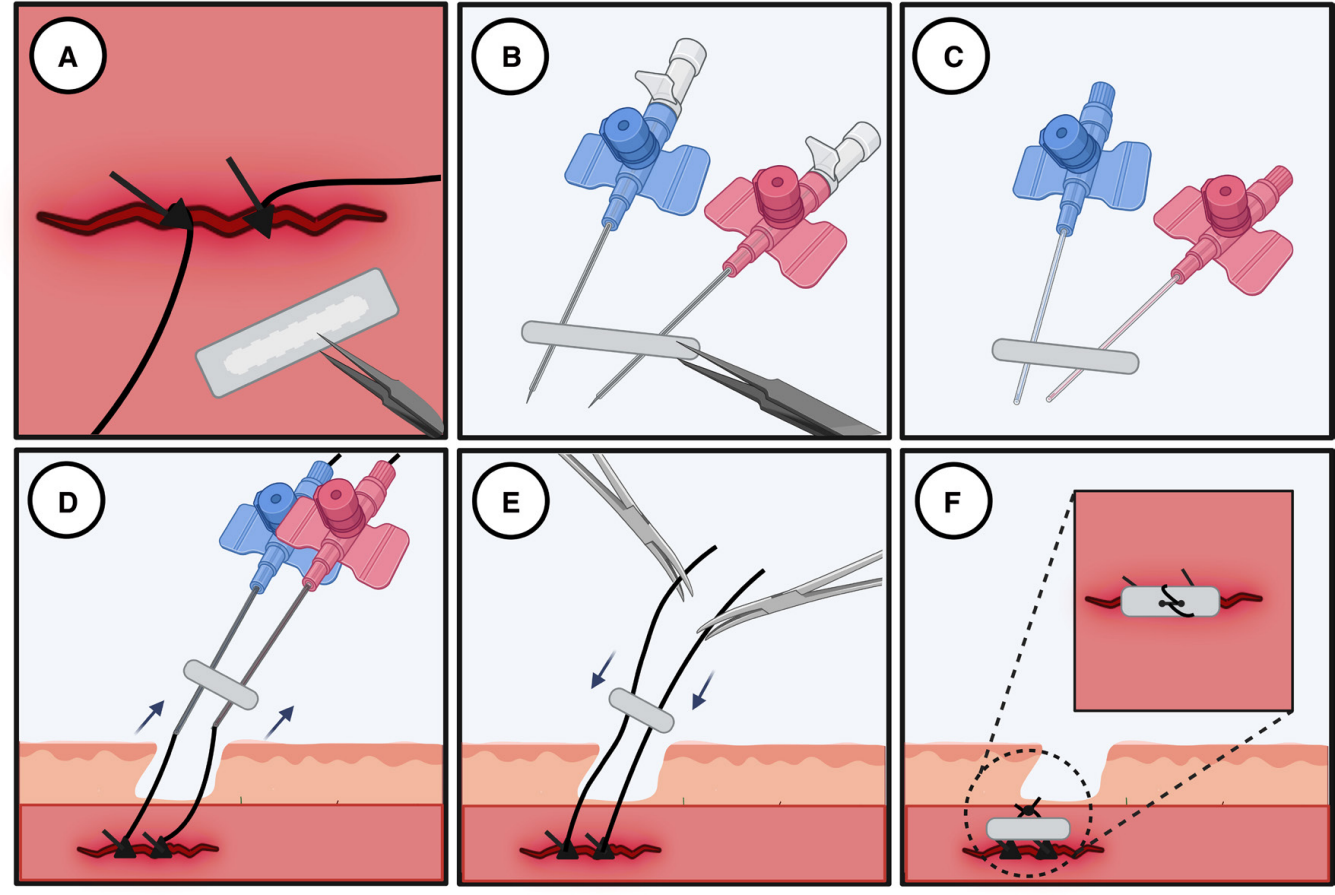
**Pledget-assisted hemostasis technique.** This technique implicates placing a nonabsorbable polytetrafluoroethylene pledget (6.5 mm×4 mm×1.5 mm; **A**) over the 2 ProGlide sutures (**B**). The pledget is then pressed down using the ProGlide knot-pusher and secured with a manually tied sliding knot (**C** through **E**). This method ensures stable alignment with the vessel wall for optimal hemostasis (**F**). Created with BioRender.com.

VCD failure can lead to acute limb ischemia (often resulting from acute thrombosis due to extended device use and dissection during access manipulations) or severe bleeding.^42^ Endovascular treatments offer a flexible, effective solution post-VCD failure for both ischemic and bleeding complications (Figure 6) and could be performed through contralateral transfemoral access or transradial access.^43^ The availability of ultra-long guidewires and 6-Fr-compatible long-shaft balloons/stents now makes transradial access more feasible for lower limb endovascular interventions. To access the descending aorta via a transradial access, an exchange-length *J*-tipped wire is used. When a tortuosity in the subclavian or aortic arch is encountered, utilizing a 90-cm sheath can improve the maneuverability of the guide catheter. A 125-cm, 6-Fr multipurpose (MP) guiding catheter is then navigated into the descending aorta over a 300-cm, 0.035″ J-tip guidewire. With this setup, selected balloons can be easily advanced to a position proximal to the access site using a 400-cm 018″ Plywire guidewire (Optimed, Norcross, GA), allowing for either hemostasis or the restoration of distal blood flow. When dealing with a residual stenosis, peripheral stent placement may be considered, and operators need to be aware that only a few stents are 6- or 7-Fr compatible. If residual thrombosis occurs, manual thrombectomy with 145-cm dedicated devices or alternatively using the 125-cm MP guiding catheter may be performed. A similar endovascular strategy is applicable in situations of continuous bleeding, particularly when ipsilateral access is missing (ie, absence of a guidewire through the access port) and introducing an additional VCD for bail-out is not feasible. In such bleeding scenarios, it is crucial to maintain balloon inflation for up to 5 minutes, potentially repeating the process multiple times to achieve hemostasis or deliver ProGlide during balloon inflation (eg, bail-out dry-seal techniques). In highly selected cases, the deployment of a covered stent may be necessary to effectively cease bleeding. It is important to note that larger-sized balloons and covered stents are not compatible with 6/7-Fr systems. Therefore, obtaining contralateral femoral access is essential in these situations. Traditionally, vascular surgery is the last option when all other bail-out techniques are unsuccessful. However, recently a hybrid bail-out technique for achieving complete hemostasis in patients with double suture-based VCD failure^44^ has been described. This technique, known as pledget-assisted hemostasis, implicates placing a nonabsorbable polytetrafluoroethylene pledget (6.5 mm×4 mm×1.5 mm) over the 2 ProGlide sutures, as described in Figure 7.

## MANAGEMENT OF LARGE-BORE VENOUS ACCESS

**Figure 8.**
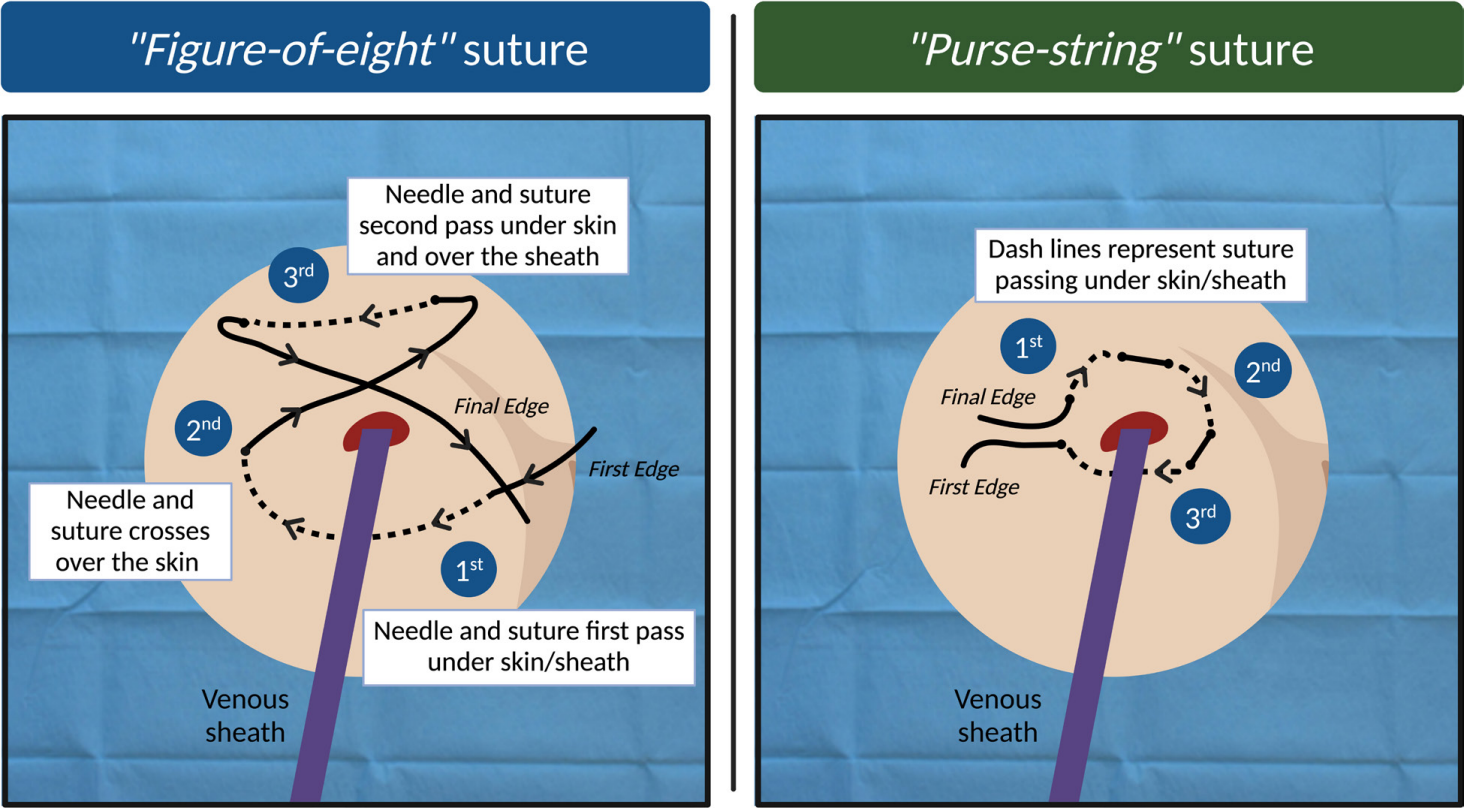
**Subcutaneous sutures techniques for large-bore transvenous transcatheter procedures.** Figure-of-eight suture (**A**); purse-string suture (**B**). Created with BioRender.com

In contrast to arterial access, the sheath size of devices used for transvenous intervention has progressively increased. Unfortunately, bleeding events and VCs are encountered even after venous catheterization, with an occurrence that ranges from between 0% and 13%.^45,46^ VCs mostly result from accidental damage to femoral artery; thus, US guidance in puncturing femoral vein is pivotal^47^ and should regarded as the gold standard technique for gaining LBVAs. Moving to the postprocedural management of the vein, traditionally, venous hemostasis was achieved through manual compression. Yet, this method could be less effective when dealing with increasing sheath size or in the presence of uninterrupted anticoagulation and prolongs immobilization time (potentially increasing the risk of deep venous thrombosis). As the main alternative, subcutaneous sutures (usually practiced using tick surgical thread) have been developed with the aim of achieving the plication of the subcutaneous tissue, which may warrant (immediately at the time of sheath/device removal) a local compression over the vein entry site. Two different subcutaneous suture techniques (Figure 8) have been proposed for the access closure in transvenous procedures requiring large-bore (up to 24-Fr) sheaths.

- Figure-of-eight (FO8) suture (also known as the z-stitch or fellow-stitch). Several studies, both randomized^48^ and meta-analysis,^49^ have compared the FO8 suture with manual compression and have demonstrated that the FO8 stopped bleeding in <60 seconds with shorter immobility time, hospital stay, and significantly fewer VCs.
- Purse-string suture. This technique has been studied in sheaths up to 24-Fr, showing significant advantages as compared with manual compression (with benefit comparable with that reported for FO8).^50^

Among VCD developed for arterial hemostasis, the ProGlide use in venous access has been proposed for sheaths up to 29-Fr OD and was approved for this use by the FDA in 2018. To date, there is limited literature evaluating VCD use in LBVA. The largest study^51^ investigating VCD use in LBVAs demonstrated that preimplantation of 1 to 2 ProGlide is safe and related with an extremely low rate of VCs. In addition, Yeo et al^52^ showed that using 2 ProGlide after transcatheter edge-to-edge repair (TEER) procedure was safe and effective, as assessed by Unied States, at 1 and 12 months. However, even with these positive results, there is still a possibility of device failure, VCs, pseudoaneurysm, and access-site infection.^45^

## CONCLUSIONS

Optimizing femoral-LBAA and LBVA management is crucial in endovascular interventions. Technical options, including optimized preprocedural planning and proper selection and utilization of VCDs, have been developed to increase safety. This review explores the comprehensive management of LBAs, from optimal vascular puncture to sheath removal. It also discusses strategies for managing closure device failure, with the goal of minimizing vascular complications.

## ARTICLE INFORMATION

### Sources of Funding

None.

### Disclosures

Drs Burzotta and Trani received speaker fees from Abbott Vascular, Abiomed, Medtronic, and Terumo. Dr Paraggio received speaker fees from Abiomed and Terumo. Dr Aurigemma received speaker fees from Abbott Vascular, Abiomed, Medtronic, Terumo, and Daiichi Sankyo. Dr Romagnoli received speaker fees from Abiomed, Abbott Vascular, and Terumo. Dr Bianchini received a research grant from Abbott Vascular. The other authors report no conflicts of interest to declare.
